# Nanoscale Characterization of V-Defect in InGaN/GaN QWs LEDs Using Near-Field Scanning Optical Microscopy

**DOI:** 10.3390/nano9040633

**Published:** 2019-04-18

**Authors:** Yufeng Li, Weihan Tang, Ye Zhang, Maofeng Guo, Qiang Li, Xilin Su, Aixing Li, Feng Yun

**Affiliations:** 1 Shaanxi Provincial Key Laboratory of Photonics and Information Technology, Xi’an Jiaotong University, Xi’an 710049, China; yufengli@mail.xjtu.edu.cn (Y.L.); tangweihan2012@163.com (W.T.); zhangye830108@mail.xjtu.edu.cn (Y.Z.); guomaofeng2011@xjtu.edu.cn (M.G.); liqiang_ynu@126.com (Q.L.); suxilin111@163.com (X.S.); liaixing@stu.xjtu.edu.cn (A.L.); 2 Solid-State Lighting Engineering Research Center, Xi’an Jiaotong University, Xi’an 710049, China

**Keywords:** V-defect, InGaN/GaN QWs, LEDs, NSOM, quantum efficiency

## Abstract

The size of the V-defects in the GaN/InGaN-based quantum wells blue light-emitting diode (LED) was intentionally modified from 50 nm to 300 nm. High resolution photoluminescence and electroluminescence of a single large V-defect were investigated by near-field scanning optical microscopy. The current distribution along the {10-11} facets of the large defect was measured by conductive atomic force microscopy. Nearly 20 times the current injection and dominant emission from bottom quantum wells were found in the V-defect compared to its vicinity. Such enhanced current injection into the bottom part of quantum wells through V-defect results in higher light output power. Reduced external quantum efficiency droops were achieved due to more uniform carrier distribution. The un-encapsulated fabricated chip shows light output power of 172.5 mW and 201.7 mW at 400 mA, and external quantum efficiency drop of 22.3% and 15.4% for the sample without and with large V-defects, respectively. Modified V-defects provide a simple and effective approach to suppress the efficiency droop problem that occurs at high current injection, while improving overall quantum efficiency.

## 1. Introduction

Gallium nitride (GaN)-related materials are widely used for optoelectronics devices, such as visible to near UV light-emitting diodes (LEDs), detectors and laser diodes (LDs). Unlike other III-V material systems, the radiative efficiency of GaN-based quantum wells (QWs) remains high, despite the presence of a high density of threading dislocations [1,2,3]. V-defects were initially considered as a contributor to the low efficiency, bad thermal stability and the large leakage current for most indium gallium nitride (InGaN)/GaN-based LEDs [4,5,6]. However, many commercial LEDs have been found to contain this V-defect, and multiple studies have put forward that the V-defect forms localized potential barriers to prevent the carriers from reaching the non-radiative recombination path [7,8,9,10,11,12,13]. The reason behind this idea has long been debated. Wu et al. [14] reported the electroluminescence (EL) of the sidewall multiple quantum wells (MQWs) at cryogenic temperature, and provided a proof of the carrier accumulation in the sidewall QWs. Broadening of photoluminescence (PL) emission has been attributed to hole injection from V-pits into c-plane MQWs [15]. From the temperature-dependent micro PL, it is speculated that carriers absorbed by sidewall QWs move to the c-plane QWs [16]. 

A theoretical model regarding the carrier injection has been proposed by simulation [17]. On the other hand, nanoscopic spectroscopy with high spatial resolution, using near-field scanning optical microscopy photoluminescence (NSOM-PL) and Cathodoluminescence (CL) were used to study the barrier fluctuation of indium gallium nitride (InGaN) epi material and the V-defect [18,19,20,21,22,23,24,25,26]. Nevertheless, there is still a lack of clear correlation between the electrical and optical characteristics of the V-defect in nanoscale. It is necessary to determine if the enhancement of radiative efficiency in the V-defect sample is due to the change of carrier transportation or the material quality, and whether or not it is related to the presence of V-defects. Direct proof regarding the carrier transportation inside the V-defects is still missing.

In this study, with the help of near-field scanning optical microscopy (NSOM) and conductive atomic force microscopy (CAFM) measurement, we were able to differentiate the optical as well as electrical properties of the V-defect from its vicinity area in nanoscale resolution, and then further identify the roles of the V-defect. Not only by NSOM-PL, we explored the possibility of NSOM-EL, and found its correlation with the current-voltage characteristics within the V-defect. We intentionally design QWs with different emission wavelengths to identify the physical depth of carrier injection. Based on that, the possible cause of efficiency “droop” (efficiency reaches a peak value in the low injection-current regime and tends to decrease as the injection current increases), which was previously attributed to carrier delocalization [27], Auger nonradiative recombination [28], spontaneous emission reduction [29], carrier leakage [30] and poor hole injection efficiency [31], are further discussed.

## 2. Materials and Methods

### 2.1. Epitaxial Growth and Chip Fabrication

**Figure 1:**
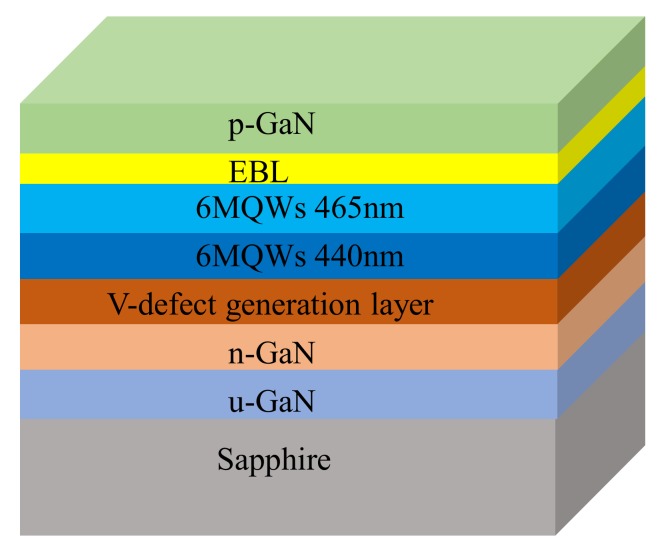
Schematics of epitaxial structure of multiple quantum wells (MQWs) light-emitting diodes (LEDs).

Light-emitting diodes (LEDs) samples were grown on regular flat (0001)-oriented sapphire substrates (Epitech Inc., Xi’an, China) by Metal Organic Vapor Phase Epitaxy (MOVPE). A schematic of the epitaxial layer structure is illustrated in Figure 1. First, a 2-μm undoped GaN layer, and a 3-μm Si-doped GaN layer were grown on the sapphire substrate. A 150–200 nm V-defect generation layer was grown at a low temperature for a large V-defect sample, while a layer with the same thickness was grown under regular growth conditions for a reference sample. The low temperature layer was supposed to serve as a function to generate the V-defect at the early growth stage, so that a large diameter V-defect can be formed during the quantum wells (QWs) growth. The active region consisting of 12 pairs of undoped GaN/InGaN multiple quantum wells (MQWs) was then grown. The first grown 6 pairs of bottom QWs were designed to emit at 440 nm, while the other 6 pairs of top QWs were designed to emit at 465 nm. Holes are known to have difficulty transporting to QWs located far away from the p-GaN due to its low mobility. In order to indirectly locate hole injection depth, we purposely design two such sets of QWs structure, hoping that the different emission wavelength can indicate the coarse location of the carrier recombination. In most real applications, QWs were intentionally grown with uniform wavelength. Thereafter, a 100-nm undoped aluminum gallium nitride (AlGaN) electron-blocking layer (EBL) was grown, followed by a 200-nm Mg-doped p-GaN layer. MQWs samples without the p-type capping layer were separately grown to obtain the geometrical information of large V-defects.

LED chips were fabricated in vertical structure, in which the sapphire substrate was removed using the laser lift-off process, and the device was bonded onto the conductive substrate. Such conductive substrate has the advantages of a large emission area and good thermal conductivity. Therefore, it has better performances than those of the lateral LED. A Ni (3 nm)/Ag (20 nm) /Pt (200 nm) p-electrode was deposited on the p^+^-GaN surface, and then the wafer was annealed at 500 °C in a nitrogen atmosphere for 3 min. Two pairs of Ti (200 nm)/Au (400 nm) were deposited as the bonding layer. The wafer was then bonded to the 2 inch Cu/W substrate at a temperature of 300–330 °C for 20 min in a vacuum. The sapphire removal process was carried out using a laser-lift-off machine from JPSA (JP Sercel Associates, Inc., Manchester, NH, USA). The repetition rate of the ArF (248 nm) laser was set at 50 Hz with a 10 ns pulse width. The laser power was 100 mW, and the moving stage velocity was 30 mm/s. After removal of the sapphire substrate, the wafer was immersed in HCl acid for 60 s to remove micro-sized metal residuals. A regular photolithography process was performed, followed by Cl_2_/BCl_3_-based inductively-coupled plasma etching to define the chip mesa area (45 × 45 mil^2^) first, and then remove the un-doped GaN layer. Ti/Al/Ti/Au (100/300/200/400 nm) was immediately deposed as the n-electrode on the exposed n-GaN surface.

### 2.2. Characterization

Atomic force microscopy (AFM) images of the sample surface were measured in tapping mode (Dimension Icon, Bruker, Karlsruhe, Germany). A Transmission Electron Microscope (TEM)-ready sample was prepared using the in-situ Focused Ion beam (FIB) lift-out technique on a Field Electron and Ion Company (FEI) Strata 400 Dual Beam FIB/Scanning Electron Microscope (SEM) (FEI, Hillsboro, Oregon, USA). Then the sample was imaged in an FEI Tecnai TF-20 Field Emission Gun (FEG)/TEM operated at 200 kV in bright-field (BF) TEM mode, high-resolution (HR) TEM mode, and High-Angle Annular-Dark-Field (HAADF) STEM mode. Regular power-dependent PL spectra were measured under room temperature (RT) with a 405-nm laser from PTI (Power Technology, USA). With a home-build micro-PL setup, the laser beam was focused down to 35 × 35 μm^2^, so that the excitation density was varied from 7.01 to 166 W/mm^2^. The spectra were measured with a spectrometer (Horiba iHR550, Jobin Yvon, Kyoto, Japan). Regular electroluminescence (EL) spectra of full LED structure chip from 2 to 400 mA in DC mode were measured under RT. The light output power (LOP) of the un-encapsulated LED chips was measured as a function of injection current using a calibrated detector, based on which the external quantum efficiency (EQE) was calculated. NSOM-PL and near-field scanning optical microscopy-electroluminescence (NSOM-EL) mapping as a function of excitation density were performed in collection-mode, using NSOM (NTEGRA, NT-MDT, Moscow, Russia) with a tip aperture size of 100 nm at RT. 

The conductive atomic force microscopy (CAFM) image was measured with the NSOM system in spreading resistance (SR) mode. Temperature-dependent PL spectra from RT to 7 K were measured in the cryogenic system OPTIDRYBL4W (Oxford Instrument, Oxford, UK) to calculate the internal quantum efficiency (IQE) of bottom and top quantum well sets, separately. 

## 3. Results

**Figure 2:**
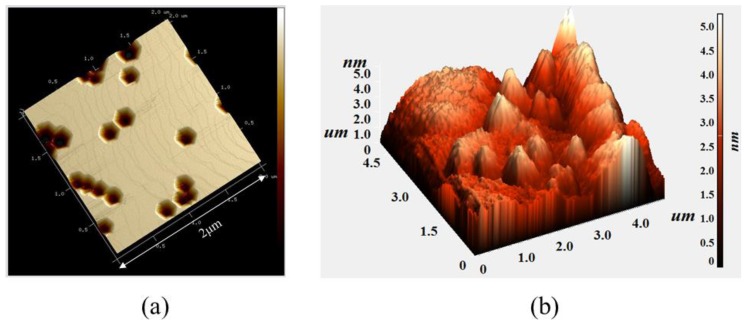
Atomic force microscopy (AFM) image of (**a**) MQWs without upper p-GaN capping layer and (**b**) full LED epitaxy with upper p-GaN capping layer for sample with large V-defects, respectively.

Figure 2a shows an atomic force microscopy (AFM) image of the MQW sample with large V-defects. The density of the large V-defects was calculated to be 1.53 × 10^8^/cm^2^, and the size is approximately 280 ± 20 nm in diameter. For a reference MQW sample without the intentionally grown V-defect, the top diameter of the individual small pit is 50 ± 10 nm (not shown). The areas of the small and large V-shaped pit regions were calculated to be about 3.8% and 17%, respectively. For a full LED structure, a sufficiently thick p-type GaN capping layer was grown to fill the V-defects, and the top surface appeared to be completely smooth. Figure 2b shows an AFM image of a p-GaN surface of the LED sample with large V-defects. The RMS was measured as 3.1 nm, showing smooth topology. Therefore, the size and the density of the V-defect do not affect the light extraction efficiency, which was excluded in the discussion of EQE and LOP below.

**Figure 3:**
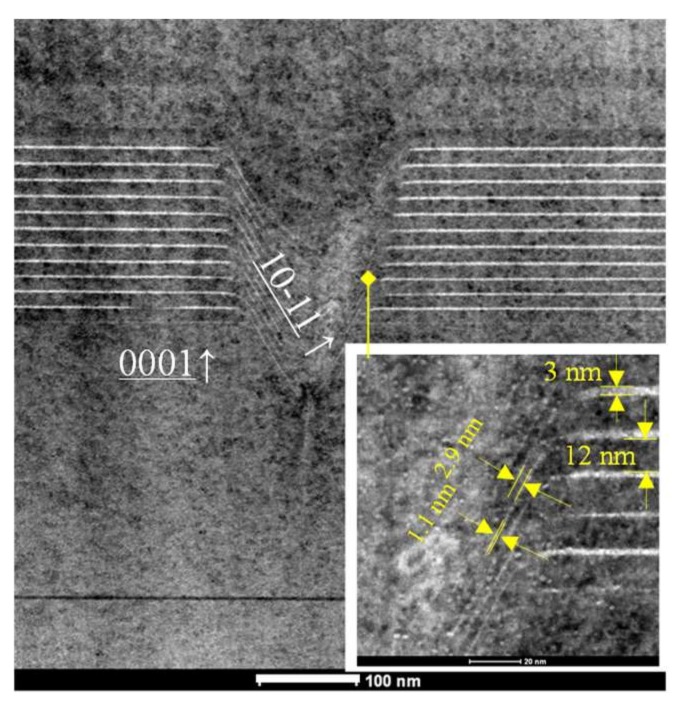
High-resolution Transmission Electron Microscope (TEM) image of a large V-defect, the inset shows the detail of side wall quantum wells (QWs) growth in the V-defect, i.e., the {10-11} plane to c-plane QW transition area.

Figure 3 shows a TEM image of a large V-defect with an individual V-shaped facet clearly distinguished. Before the formation of the V-defect, threading dislocations (TDs) were formed during epitaxy growth of the low-temperature pit generation layer. V-defects are known to generally nucleate on the TDs across the first InGaN QW layer, due to strain relaxation [32,33]. The flat MQWs grew in the (0001) direction and the inclined MQWs V-defect grew along the {10-11} orientation. The TEM result shows that the large V-defect, initiated by a threading dislocation, starts to grow before the first pair of QWs, as designed, and their depth is about 150–200 nm. The thickness of c-plane-oriented QW and QB were measured as 3 nm and 12 nm, respectively. The QW and QB grown along {10-11} inside the V-pits was measured as 1.1 nm and 2.9 nm, respectively. The thickness of inclined QWs is almost 1/3–1/4 that of the c-plane QWs, which is similar to previous researches [7].

**Figure 4:**
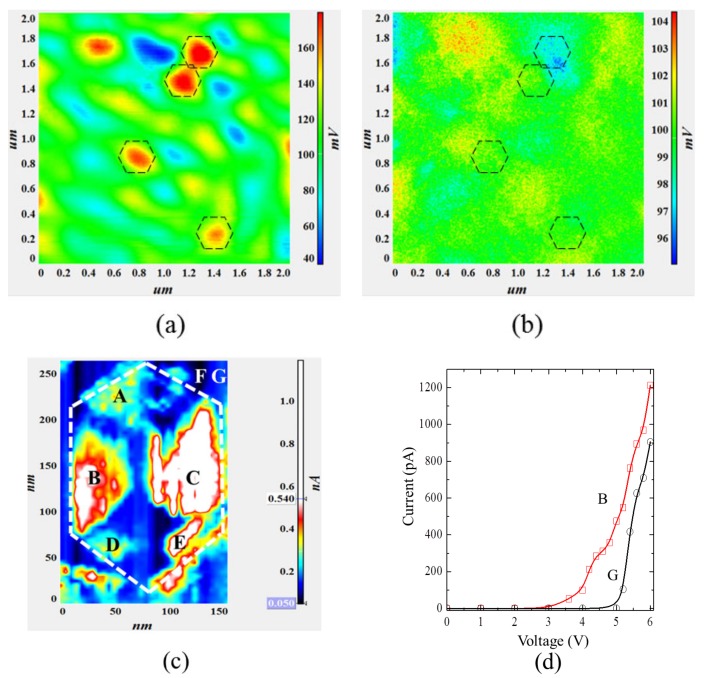
(**a**) near-field scanning optical microscopy-electroluminescence (NSOM-EL) and (**b**) near-field scanning optical microscopy photoluminescence (NSOM-PL) mapping for large V-defect sample in the scale of 2 × 2 μm^2^, (**c**) conductive atomic force microscopy (CAFM) mapping around a large V-defect, (**d**) I-V characteristics of point G (outside V-defect) and B (inside V-defect).

The growth of the p-GaN layer makes it difficult to identify the V-defect by scanning the surface morphology. Therefore, NSOM-PL and -EL scans were employed to find the location of this V-defect by NSOM. Since the V-defect is about 300 nm in diameter and the distribution density is approximately 1.53 × 10^8^/cm^2^, an area of 2 × 2 μm^2^ was chosen to make sure one or more V-defects had been included. Figure 4a,b show the NSOM-EL and NSOM-PL intensity mapping, respectively. The intensity variation across the scanning area is relatively small for NSOM-PL mapping, indicating uniform carrier injection and recombination during optical pumping [34,35,36]. But in NSOM-EL mapping, one can see a clear contrast between bright and dark regions. Several highlighted zones, marked as hexagonal shapes, show a much higher intensity than their neighborhood. Those areas do not seem to have a direct correlation to the surface roughness as shown in the AFM image. Apparently, those areas behave differently under electrical injection compared to optical pumping. Conductivity mapping was performed by CAFM. Figure 4c shows the current mapping under constant voltage mode in one bright area from Figure 4a. The current injection distribution was visualized with a resolution of 30 nm, a much better spatial resolution compared to NSOM. The current mapping shows a clear hexagonal pattern with six facets, implying the injection paths mainly located around the {10-11} facet. The currents that were measured at points A to G are 300, 550, 900, 300, 650, 50, and 150 pA, respectively. The maximum measured current (brightest area) was close to 1000 pA, which is equivalent to 300 mA/mm^2^. The minimum current (darkest area) was measured as 50 pA, resulting in a max/min contrast ratio of 20:1. In fact, the current injection found from the inclined {10-11} facet inside the V-defect is nearly 20 times higher than the c-plane QWs, indicating that more carriers choose to inject QWs from large a V-defects area, and recombine there. Figure 4d shows the current-voltage characteristics of the location G (outside) and B (inside) of a single V-defect. The turn-on voltage is about 5 V outside and 3.4 V inside the V-defect area. These numbers are relatively large, compared to a conventional LED chip (e.g., 2.1 V), due to the imperfect ohmic contact between p-GaN and the CAFM tip. At small currents, the I-V curve inside V-defect shows a smaller slope than the outside of the V-defect, indicating a shunt path. At a large current, both I-V curves show similar slope, indicating the magnitude of the serial resistance is the same. It implies that the current injection mechanism is different between the inside and outside of the V-defect at a small current region (0–0.05 A/mm^2^). To some extent, this result is similar to the higher conductivity of the V-defect observed in AlGaN/GaN heterostructures, where V-defects are believed to provide preferential conduction paths to the AlGaN/GaN interface [37]. However the contrast of the current scanning area is larger, probably due to the much larger size of our V-defect. Both positions show very small reverse current, approximately 4 pA, (250 times smaller than the forward current), up to −6 V. At higher current density, the mapping contrast decreases due to the large background light caused by light propagation under the waveguide effect. The high injection current, as well as the bright EL intensity under electrical bias seems to rule out the possibility that the V-defects may exhibit an energy barrier or electrical passivation to prevent carriers from reaching the V-defects.

**Figure 5:**
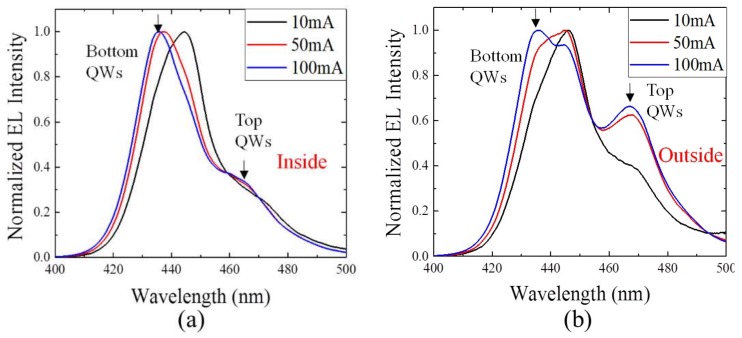
NSOM-EL spectra of area (**a**) inside and (**b**) outside of a V-defect at 10, 50 and 100 mA, respectively.

**Figure 6:**
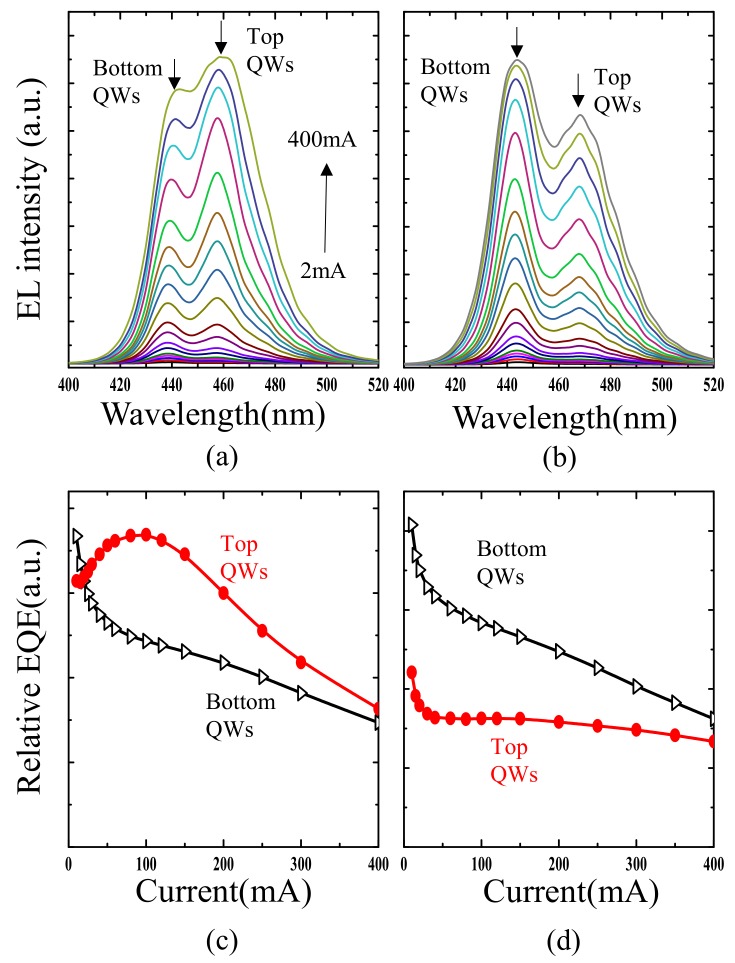
Current-dependent regular EL spectra and EQE vs. current for (**a**,**c**) reference sample (**b**,**d**) large V-defect sample.

Figure 5a shows normalized NSOM-EL spectra interior and outside of the V-defect at 10, 50 and 100 mA, respectively. The short (440 nm) and long (465 nm) emission correspond to the bottom and top set of QWs, respectively. It was found that inside the V-defect, the bottom QWs’ emission (440 nm) always dominates, and the emission from the top set (465 nm) is almost absent. Outside the V-defect the peak intensities of top QWs rise significantly at 50 and 100 mA (Figure 5b) but they are still lower than those of the bottom QWs. When moving further outward, the top QWs’ emission becomes even stronger until the measurement was affected by another V-defect next to it. This phenomenon indicates that the bottom QWs set in the V-defect has higher carrier injection than that outside of the V-defect. The regular EL measurement, in which a much larger area was included, shows a similar dominance switching effect. Figure 6a,b show the regular EL spectra for the sample without and with the V-defect from 2 to 400 mA, respectively. The bottom QWs’ emission in the large V-defect sample is obviously larger than the emission of the top QWs. The opposite is true for the reference sample. When plotting the relative EQE as a function of current, one can tell that for both samples, the bottom QWs show the regular “droop” effect. Its emission intensity shows a monotonic decrease, but those top QWs behave differently. For the reference sample, the top QWs’ emission always has a higher intensity than that of the bottom QWs, and it reaches a local EQE maximum close to 100 mA (Figure 6c). For the large V-defect sample the top QWs emission is weaker, and does not show much “droop” effect (Figure 6d). 

**Figure 7:**
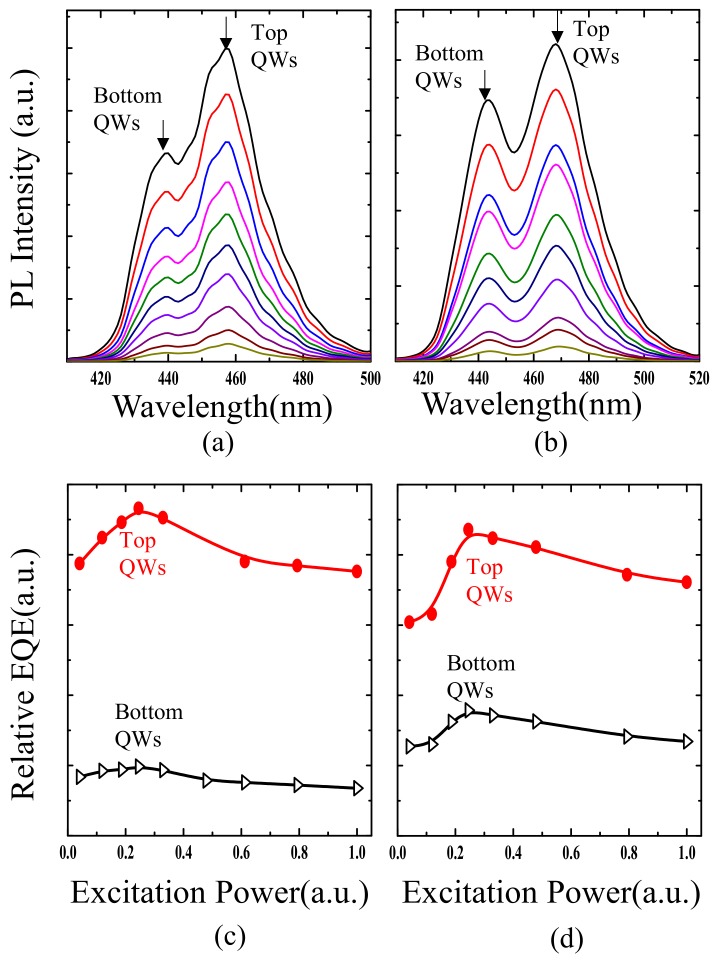
Power-dependent PL spectra of sample A (**a**) and B (**b**), excitation power density-dependent relative quantum efficiency for sample A (**c**) and B (**d**).

**Figure 8:**
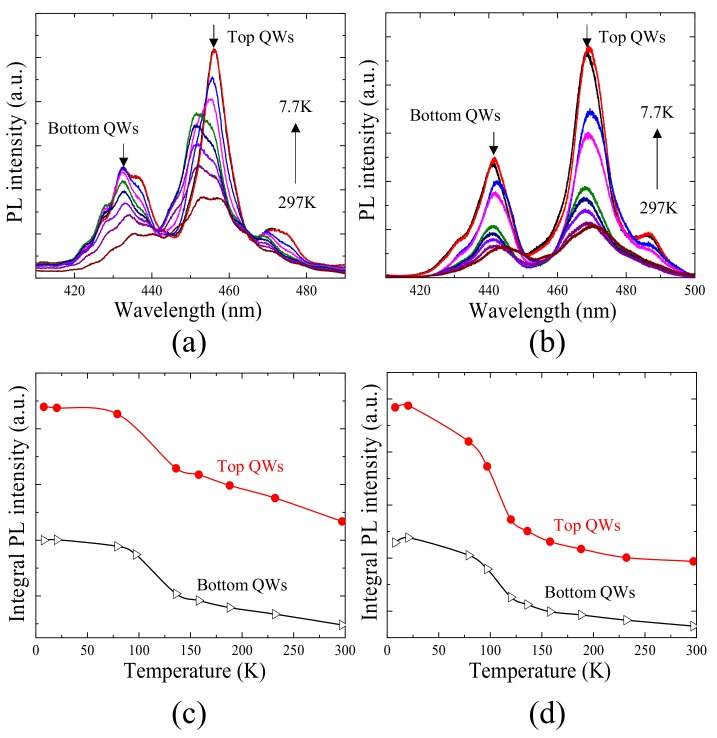
Temperature-dependent PL spectra for (**a**) reference sample and (**b**) sample with large V-defects. Integrated PL intensity vs. temperature for (**c**) reference sample and (**d**) sample with large V-defects.

The PL spectra show behavior much different from regular EL and NSOM-EL. Figure 7a,b show the PL spectra from 7.01 to 166 W/mm^2^ for the reference and for the large V-defect sample, respectively. For both samples the emission from the top QWs is always higher than that of the bottom QWs. Figure 7c,d show the relationship between the quantum efficiency as a function of the excitation power density for top and bottom sets of QWs, respectively. The two efficiency curves look very much alike, and both show the conventional “droop” effect at the same level of excitation density. When we switched the excitation and detection direction, e.g., the samples were excited from the p-GaN side, and detected from sapphire side, the same results were observed. It indicates almost uniform carrier generation in both top and bottom QWs during optical pumping. It also implies that the difference in intensity is not from the QW absorption of laser near the pumping source. The reason why the top QWs’ intensity is higher than the bottom QWs, was further investigated by a temperature-dependent PL measurement (Figure 8). IQE of each set of QWs were calculated (shown in Table 1). The IQE of the top QWs is always higher than the bottom QWs. Such results are probably due to the fact that the QWs that were grown later have better material quality than the QWs that were first grown. The IQE of top QWs in a large V-defect sample is reasonably smaller than that of the reference sample, due to the introduction of a large V-defect and the deterioration of the crystal quality due to lattice relaxation.

**Figure 9:**
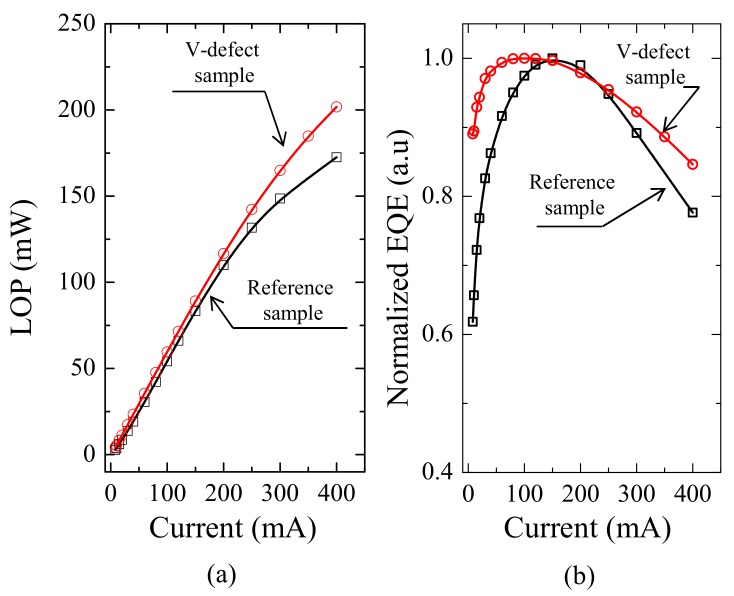
(**a**) LOP and (**b**) normalized EQE as a function of current for V-defect sample (circle) and reference sample (square).

Figure 9a shows the LOP of fabricated dies (45 × 45 mil^2^). LOP measured at 400mA for reference and large V-defect sample were 201.7 and 172.5 mW, respectively. The LOP is considered to be relatively low compared to report value, such as 520 mW at equivalent current reported by the group from LG Korea [38]. However this is because we did not optimize the light extraction efficiency, and did not encapsulate the die. Given the fine tuning of the light extraction feature, such as roughening and proper refractive index match encapsulation, the LOP can be expected to be more than twice the amount measured above. Figure 9b shows the normalized EQE as a function of the current. At 400 mA the EQE of the reference sample decreased by 22.3% from its maximum value, similar to the recent report droop value of roughly 20% [38]. The V-defect sample shows a much smaller “droop” effect: EQE only decreased by 15.4% at the same current. Compared to Figure 6d, the small variation of EQE at large currents may benefit from the small change of EQE of the top set of QWs in the large V-defect sample. The median reverse current at −8 V was measured as 0.008 mA for large V-defect samples. These facts indicate that the large V-defects do not particularly serve as electrically critical defects, nor do they predominantly facilitate non-radiative recombination.

So far, we have measured NSOM-EL, NSOM-PL, regular EL and PL. We found that for the sample with large V-defects, the bottom emission dominates the EL while the top emission dominates the PL. It indicates that under current injection, the bottom QWs emit more photons than the top QWs, even though its IQE is lower than the top ones. The NSOM-EL inside the V-defect shows similar results as the regular EL, and suggests that such bottom QWs’ emission dominance is significantly correlated with the presence of the large V-defects. The CAFM results verify that the V-defect is more conductive, and may provide a shunt path for current injection. Therefore, a more uniform carrier distribution in all QWs should be expected, so that the radiative recombination efficiency of the carriers in each QW is higher. This mechanism leads to a significant improvement in total LOP, and less droop of EQE.

**Figure 10:**
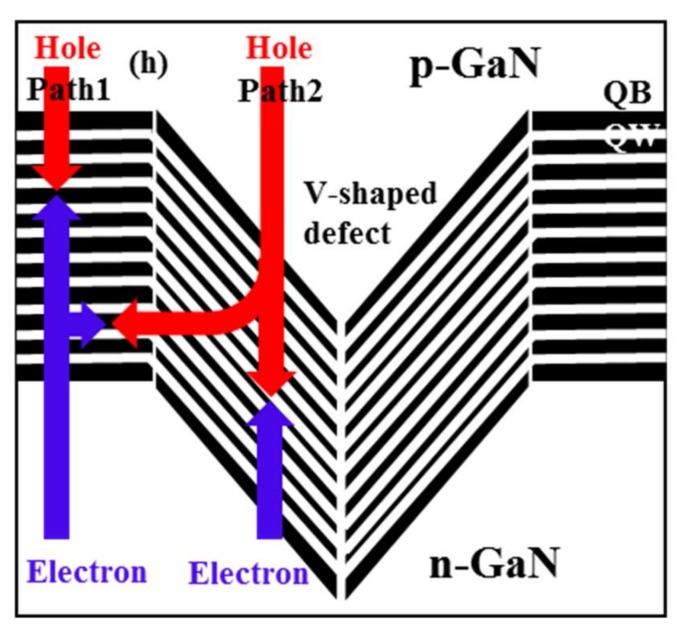
Schematic of the possible current path in large V-defect region and V-defects-free regions.

Figure 10 shows a schematic of the possible current path in large V-defect regions and V-defects-free regions. Path 1 stands for the current injected from the top QWs into the V-defects-free area. Due to the low mobility of the holes, only the top set of QWs participate in carrier recombination. Path 2 represents current injection through the V-defects, bypassing one or several of the top QWs.

Path 2 acts as a shunt path for carrier injection, while these bottom QWs’ emission dominates. Carrier recombination takes place in both top and bottom sets of QWs, thus reducing the carrier concentration in each QW. Therefore, the large V-defects sample shows a smaller “droop” effect than the reference sample. With increasing current, path 1 starts to compete, and the additional carriers are eventually forced to flow through path 1, and recombine in some or all of the top QWs.

## 4. Conclusions

The paper provides direct evidence of enhanced hole injection from the inclined sidewall of a large V-defect. CAFM measurement shows large V-defect servers as a shunt path, and offers an alternative to carrier transportation. NSOM enable us to measure nanoscale EL, which exhibits dominance bottom QWs’ emission, and the droop near large a V-defect is much less, compared to its vicinity. More QWs located far away from p-GaN were found included in the radiative recombination process, and the resulting uniform carrier distribution enhances the total LOP, and reduces the droop effect of the device. IQE measurements show that introducing large V-defects does not degrade the crystal quality. The implementation of the modified V-defect provides a promising approach to improve the efficiency of GaN/InGaN-based QWs’ LEDs operating at high current injection.

## Figures and Tables

**Table 1:** IQE of each sets of QWs.

Sample	IQE of Bottom QWs	IQE of Top QWs
Reference	0.240	0.532
Large V-defects	0.312	0.410
